# Diversity and Above-Ground Biomass Patterns of Vascular Flora Induced by Flooding in the Drawdown Area of China's Three Gorges Reservoir

**DOI:** 10.1371/journal.pone.0100889

**Published:** 2014-06-27

**Authors:** Qiang Wang, Xingzhong Yuan, J.H.Martin Willison, Yuewei Zhang, Hong Liu

**Affiliations:** 1 Key Laboratory of Freshwater Fish Reproduction and Development (Ministry of Education), Key Laboratory of Aquatic Science of Chongqing, School of Life Science, Southwest University, Chongqing, China; 2 State Key Laboratory of Coal Mine Disaster Dynamics and Control, Chongqing University, Chongqing, China; 3 College of Resources and Environmental Science, Chongqing University, Chongqing, China; 4 College of Architecture and Urban Planning, Chongqing University,Chongqing, China; 5 School for Resource and Environmental Studies, Dalhousie University, Halifax, Nova Scotia, Canada; Institute of Botany, Chinese Academy of Sciences, China

## Abstract

Hydrological alternation can dramatically influence riparian environments and shape riparian vegetation zonation. However, it was difficult to predict the status in the drawdown area of the Three Gorges Reservoir (TGR), because the hydrological regime created by the dam involves both short periods of summer flooding and long-term winter impoundment for half a year. In order to examine the effects of hydrological alternation on plant diversity and biomass in the drawdown area of TGR, twelve sites distributed along the length of the drawdown area of TGR were chosen to explore the lateral pattern of plant diversity and above-ground biomass at the ends of growing seasons in 2009 and 2010. We recorded 175 vascular plant species in 2009 and 127 in 2010, indicating that a significant loss of vascular flora in the drawdown area of TGR resulted from the new hydrological regimes. *Cynodon dactylon* and *Cyperus rotundus* had high tolerance to short periods of summer flooding and long-term winter flooding. Almost half of the remnant species were annuals. Species richness, Shannon-Wiener Index and above-ground biomass of vegetation exhibited an increasing pattern along the elevation gradient, being greater at higher elevations subjected to lower submergence stress. Plant diversity, above-ground biomass and species distribution were significantly influenced by the duration of submergence relative to elevation in both summer and previous winter. Several million tonnes of vegetation would be accumulated on the drawdown area of TGR in every summer and some adverse environmental problems may be introduced when it was submerged in winter. We conclude that vascular flora biodiversity in the drawdown area of TGR has dramatically declined after the impoundment to full capacity. The new hydrological condition, characterized by long-term winter flooding and short periods of summer flooding, determined vegetation biodiversity and above-ground biomass patterns along the elevation gradient in the drawdown area.

## Introduction

The Three Gorges Dam (TGD) in China, 2309 meters long and 185 meters in height [1], is the largest dam in the world [2]. The dam was initiated in 1994 and its first impoundment was conducted in 2003 with a water level rising of 60 m above former riverbank of the Yangtze River. When the water was raised to the ultimate planned level of about 175 m above sea level in 2010, the TGD filled the Three Gorges Reservoir (TGR), which has a surface area about of 1080 km^2^. In order to decrease sediment deposition and prolong the operational life of the TGR, water levels behind the TGD were designed to rise highest in winter (∼175 m above sea level) and decline lowest during the summer rainy season (∼145 m above sea level), thereby producing a drawdown area of about 348.93 km^2^ between the low-water and high-water marks [3].

As expected, reservoir impoundment and the associated water-level fluctuation has resulted in the inundation and alteration of riparian vegetation, and has also introduced other environmental problems such as bank collapse, water eutrophication and greenhouse gas emissions. Problems like these have become global challenges due to increasingly extreme weather events resulting from climate change [4], [5] and the rising number of dams [3]. The effects of hydrological alterations need to be better understood in order to evaluate the impacts of dams and this is also necessary for successful ecological restorations [6]. In the past two decades, much research has been conducted to study the influence of damming on riparian vegetation, but most of the research has been focused on the downstream reaches of rivers [7]. Relatively few studies have addressed the influence of reservoir impoundment or regulation on vegetation in the drawdown area (e.g. [8], [9]). Floods in these previous studies mostly took place in spring or summer, and the durations were normally less than three months [10]–[12].

The most significant difference between the hydrological regime of the TGR and other big reservoirs with similar size in the world is its regular annual winter impoundment period as long as half a year (Table 1). Hydrological observations have also shown that there are significant water level fluctuations brought by summer rains during its drawdown period. However, before the impoundment of the TGR, little is known about the effects of its hydrological regime on the drawdown area vegetation.

**Table 1:** Comparison of the Three Gorges Reservoir and other large reservoirs with similar size in the world.

Reservoir	Three Gorges Reservoir	Itaipu Reservoir	Gariep Lake	Sakakawea Lake	Mead Lake	Powell Lake
Country	China	Brazil & Paraguay	South Africa	America	America	America
Impounds	Yangtze River	Paraná River	Orange River	Missouri River	Colorado River	Colorado River, Escalante River & San Juan River
Full water volume (km^3^)	39.3	29.0	53.4	30.0	32.2	30.0
Height of dam (m)	185	196	88	64	221	220
Generally season with high water level	mid Sep to next May	irregular	summer	summer	Jan-Mar	Jun-Sep
Generally annual duration of high water level	more than half year	irregular	less than three months	irregular	less than three months	less than three months
Generally annual water level magnitude (m)	30	2	10	3∼5	6∼12	10∼15
Reference of water regime	see Fig.2	[48]	[49]	[50]	[51]	[52]

Information of reservoirs size and location were searched on *Wikipedia* (http://en.wikipedia.org/wiki/Main_Page).

In this study, we hypothesize that the hydrological regime of the TGR will alter the vascular flora and the vegetation patterns in its drawdown area. Our specific objectives were (1) to examine the change of vascular flora richness and composition induced by impoundment of the TGR and (2) to investigate the effects of the hydrological regime on the vegetation patterns in its drawdown area. Here we report two consecutive years of assessing plant communities in the drawdown zone as a follow-up to an earlier initial assessment of these communities [13]. We hope this work can supplement the sparse empirical database of the effect of winter reservoir impoundment on the drawdown area flora and be useful for the management, long-term biodiversity research and vegetation restoration of the TGR.

## Materials and Methods

### Study areas

**Figure 1:**
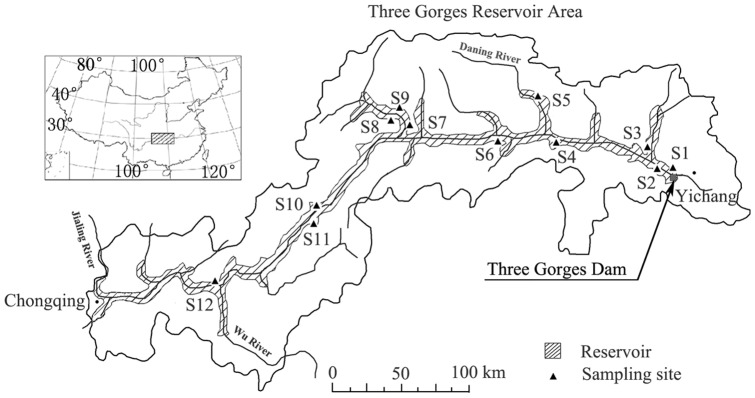
Spatial distribution of the twelve sites sampled in 2010. The sites sampled in 2009 were mostly the same and were mapped previously [13]. The Three Gorges Reservoir Area is a combination of all the counties around the reservoir.

Details of the study area have been described previously [13] and are summarized here. The drawdown area of the Three Gorges Reservoir (DATGR) stretches from the TGD in Hubei province to Jiangjin District in western Chongqing municipality (Fig. 1). Nearly 48% of the drawdown area lies in the valleys of the Yangtze and Jialing rivers [14], the latter being the largest branch of the Yangtze River in the TGA. These valleys are characterized by subtropical humid monsoonal climatic conditions, with annual mean temperature of 16.7–18.7°C. Summer is very hot with about 28 days annually having daily average temperature of 35°C or higher. Annual precipitation is 993–1242 mm and 70% of it falls during May to September. Usually there is a period of summer drought from mid-July to mid-August. The most common soil types are described as paddy soil, purplish soil, red soil and yellow soil.

In 2009 and 2010, twelve sites in the DATGR were used for vegetation community surveys (Table 2, Fig. 1). Both the TGR and its drawdown area belong to China Three Gorges Corporation. Environmental impact of any construction activities (e.g. bridge, factory, dock) here would be carefully assessed by the corporation, but no specific permissions were required for scientific survey. There were no endangered or protected species in those sampling sites. The surveys were conducted during August to early September so that we could find which species have produced seeds and estimate the total biomass of submerged vegetation prior to the re-filling of the reservoir. Six sampling sites were located beside the Yangtze River mainstream; while five sites were associated with tributaries of the main river and one site (S10) was in the drawdown area of an island in the Yangtze River. These sampling sites previously contained small woods, shrubby areas, floodplain, paddy field, dry land or village land. The sampling sites were selected with the intent of including as many as possible of the previous major land-use categories and remnant plant communities.

**Table 2:** General environmental characteristics of the twelve sampling sites in the drawdown area of the Three Gorges Reservoir.

Sampling site	Location	Longitude/latitude	Slope	Pre-dam land use
S1	Taipingxi in Zigui county	30°51′N, 110°59′E	25°	Dry land with a few woods
S2	Xiaoxingtan in Xiling Gorge	30°56′N, 110°46′E	34°	Shrubs and dry land
S3	Xiangxi River Estuary	30°58′N, 110°45′E	15°	Dry land
S4	Luyou Hole in Wu Gorge	31°03′N, 109°54′E	48°	Shrubs
S5	Dachang in Wushan County	31°15′N, 109°49′E	20°	Dry land
S6	Baidicheng in Qutang Gorge	31°02′N, 109°34′E	25°	Shrubs
S7	Shuangjiang in Yunyang County	30°56′N, 108°41′E	30°	paddy field
S8	Baijia Stream in Kaixian County	31°08′N, 108°33′E	15°	Rice paddy and dry land
S9	Laotudi in Kaixian County	31°09′N, 108°34′E	10°	Paddy field
S10	Huanghua Island in Zhongxian County	30°19′N, 108°05′E	20°	Paddy field
S11	Dongxi Stream in Zhongxian County	30°16′N, 108°04′E	18°	Paddy field
S12	Baiyansi in Fuling County	29°43′N, 107°23′E	25°	Dry land and village land

### Sampling methods

At each sampling site, we placed three transects separated by intervals at least 20 m from the waterline (145 m) to 172 m above sea level, which was the highest water level in the winters of 2008 and 2009. We divided each transect into six elevation zones at 5-m height intervals (zone I: 145∼150 m, zone II: 150∼155 m, zone III: 155∼160 m, zone IV: 160∼165 m, zone V: 165∼170 m and zone VI: 170∼172 m). At each elevation zone of each transect, dominant vegetation was selected and a 1 m×1 m plot was used to estimate the percentage cover of total vegetation and of each species. Within each plot the following were also recorded: abundance, height and phenophase of species, land use before the construction of the TGR, and substrate. Substrates were cataloged as follows based on predominant particle size and measured in field using vernier caliper: (1) clay, (2) silt (<0.05 mm), (3) sand (0.05–2 mm), (4) gravel (2 mm∼16 mm), (5) pebble (16 mm∼64 mm), (6) cobble (64 mm∼256 mm), (7) boulder(>256 mm).

Slopes of sampling sites were measured using a circumferentor. Any species outside the plots but lying in each elevation zone were also recorded to get total vascular flora richness. For taxonomy and life form, we referred to the *Chinese Virtual Herbarium* (http://www.cvh.org.cn/cms/), a database of the flora of China. The approximate age (newly germinated, one year or more than two years) of trees and shrubs were estimated based on height and trunk diameter. Above-ground biomass of vegetation of each plot was harvested and weighed after drying at 75°C for 24 h in the laboratory. In each plot, a topsoil column (Ф = 5 cm, depth = 5 cm) was sampled using a soil cutting ring and its wet and dry mass (drying at 75°C for 48 hours) was weighed to an accuracy of 0.1 g. Soil moisture content of topsoil was calculated as:

Soil moisture content  =  (Wet mass - Dry mass)/Wet mass×100%.

### Hydrological regime

**Figure 2:**
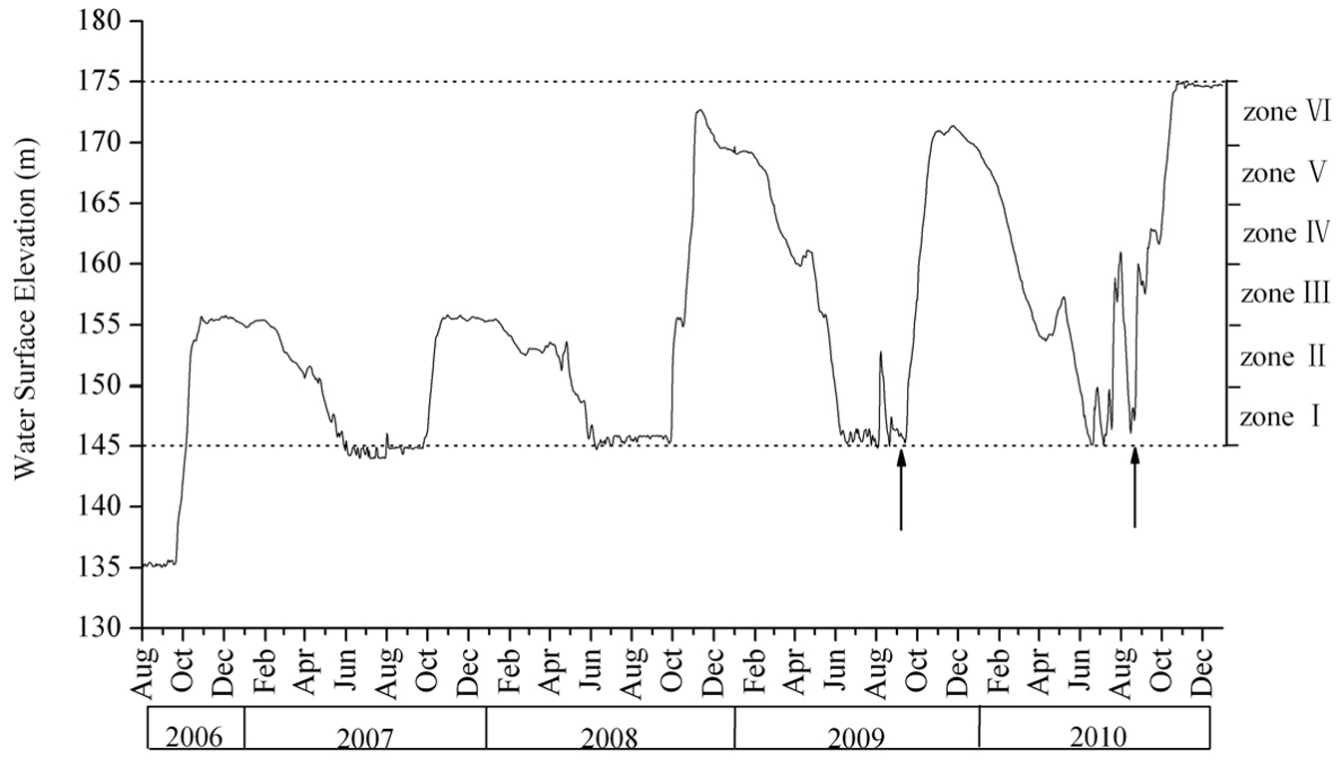
Water level of the Three Gorges Reservoir between August 2006 and December 2010. Data originate from the daily records of the Three Gorges Dam hydrology station (http://www.ctgpc.com.cn/index.php). The vertical arrows indicated the period for field sampling.

Before the impoundment of the TGD, the hydrological regime the Yangtze River was similar to most other rivers in East Asia influenced by monsoon, with floods and higher water level in summer and lower water level in winter. Since the construction of the TGD, the hydrological regime behind the dam was changed. In November 2003, the water level was raised to ∼135 m above the sea level and was maintained at that level with slight fluctuations for almost three years. Subsequent changes in the water level in the reservoir, from August 2006 to December 2010, are shown in Figure 2. In late September 2006, the annual cycle of raising and lowering the water level was started, beginning with an experimental rise to 156 m, following by lowering to 145 m in summer 2007. The winter levels in 2008 and 2009 were about 172 m, with summers again at the standard 145 m. In general, the high water periods lasted more than eight months (mid September to May), varying from year to year by a few days.

Because the raising and lowering of water level takes some time to achieve, the duration of winter submergence declines with elevation (Fig. 2). In the winters of 2008 and 2009, zone I was inundated throughout the impoundment period; zone II was submerged for more than 200 days; zone III was submerged for 194 and 157 days in 2008 and 2009, respectively; zone IV was inundated for about 4 months in both winters; zone V was submerged for about 3 months; while zone VI experienced only a few days of submergence in both winters (Table 3). In the summers of 2007 and 2008, there was virtually no flooding (Fig. 2) as a result of relatively little precipitation in the Three Gorges Reservoir Area (TGRA). In 2009 and 2010, summer flooding raised the water to above 150 m and 160 m above the sea level, respectively. The summer flood in 2010 was particularly prolonged with the water level at or above 155 m for 15 days.

**Table 3:** Submerged duration along the elevation of the drawdown area of the Three Gorges Reservoir in 2008 and 2009.

Elevation zone	Elevation range (m)	Submerged duration (d)			
		2008 summer	2008∼2009	2009 summer	2009∼2010
I	145∼150	13	243	53	257
II	150∼155	6	234	26	239
III	155∼160	-	194	15	157
IV	160∼165	-	130	4	128
V	165∼170	-	101	-	88
VI	170∼172	-	14	-	15

Impounding periods of the Three Gorges Reservoir generally last from October to June next year with several days' advance or postponement.

### Data analysis

In order to examine the effect of flooding on vascular flora in the drawdown area, we examined the absolute number and relative percentage of different life forms in the various elevation zones. Species richness, Shannon-Wiener Index and above-ground biomass in plots were employed as indicators for assessing the influence of the duration of submergence. For each indicator, data from the three plots in the same elevation zone of individual sampling sites were averaged. The differences of the averages of twelve sampling sites between six elevation zones were analyzed by one-way ANOVA. If significant differences were detected, Duncan's multiple range test was used to conduct multiple comparisons. If the assumption of homogeneity of variance was not met, the Kruskal–Wallis *H* test was employed to reveal the effects of elevation on variance. If there were significant differences, we used the non-parametric Mann-Whitney *U* test to investigate differences in indicators across elevation zones. Spearman's rank correlation coefficient (*r_s_*) was employed to discover the relationship among species richness, Shannon-Wiener Index, above-ground biomass and environmental factors. These analyses were performed with the SPSS 15.0 statistical package. Differences were considered statistically significant at *P*<0.05.

Relationships between flora and environmental parameters were investigated by Canonical Correspondence Analysis (CCA) using CANOCO 4.5 [53]. As two environmental parameters, duration of submergence in previous winter and elevation, were highly correlated according to Spearman's rank correlation coefficient, only the former was used in CCA. Significance of the first two ordination axes was assessed by Monte Carlo permutation testes (9999 permutations).

## Results

### Variation of floral composition

In 2009, 175 taxa belonging to 58 families were identified [13]. Members of the family Gramineae were most quantitatively abundant with 26 species, followed by Compositae and Cyperaceae with 21 and 14 species, respectively. The average species richness of each sampling site was 40 taxa with much site-to-site variation. S9, former paddy field located in a small valley in Kaixian County, contained the greatest richness (95 taxa) while S10, another paddy field in Huanghua Island in the mainstream of the Yangtze River, had the least (19 taxa). By contrast in 2010, we recorded 127 taxa of 44 families. Compositae was the most abundant family in terms of diversity with 20 species, followed by Gramineae and Cyperaceae with 18 and 13 species, respectively. More than half of the families were represented by only one species. The average species richness of each sampling site was 30 taxa and site-to-site variation was less than in 2009. Although S9 lost half of its taxa, it had the highest species richness (54 taxa) while S2 and S12 had the lowest species richness (23 taxa).

There was less variation between the two surveys with respect to life form than species diversity despite the total richness of vascular flora in the sampling sites having decreased by 48 species in 2010 compared with 2009. Therophytes were the dominant life form in both years, contributing 49.7% of total flora taxa in 2009 and 49.6% in 2010 (Table 4). Year-to-year variation of other life forms was generally less than 2%, with the exception of phanerophytes which decreased from 17.1% of the total in 2009 to 12.6% in 2010. In 2009 we found 29 aquatic taxa. Three-quarters of these aquatic species were restricted to an uncultivated paddy field at site S9. This lay in a valley with sufficient water resources and soil wetness for survival of aquatic flora. Most other sites appeared to be too arid for aquatic species. In 2010, there were 20 aquatic species in these sampling sites.

**Table 4:** Comparison of the absolute number and percentage of each life form category in the drawdown area before impoundment by the Three Gorges Dam and after impoundment in both 2009 and 2010.

Life form	No. of sp.		Percentage (%)	
	2009	2010	2009	2010
Phanerophytes	30	16	17.1	12.6
Chamaephytes	14	11	8.0	8.7
Hemicryptophytes	16	14	9.1	11.0
Cryptophytes	28	23	16.1	18.1
Therophytes	87	63	49.7	49.6
Total	175	127	100	100

### Flood-tolerant perennial plants

In 2009, we recorded 35 species of trees and shrubs [13], but 26 of these species were present only as seedlings or adventitious entities. For this reason it was not clear that they were truly tolerant of winter flooding. By contrast, in 2009 the following eight woody plant species were either widely distributed in the drawdown area or there were many woody shoots that were more than two years old: *Vitex negundo* L., *Morus alba* L., *Sapium sebiferum* L. Roxb., *Glochidion puberum* L. Hutch., *Rhus chinensis* Mill., *Melia azedarach* L., *Pterocarya stenoptera* DC. and *Trema levigata* Hand.-Mazz. We initially expected that these eight species would be potential candidates for vegetation restoration in the drawdown area because they had exhibited tolerance of winter flooding [13], but in 2010 we recorded 16 woody plant species in and around our sampling sites and total established sites number of the eight woody plant species listed above were dramatically depressed (Table 5). Two perennial weeds, *Cynodon dactylon* (L.) Pers. and *Cyperus rotundus* L., widely distributed in zone I, exhibited remarkable tolerance to submergence under more than 20 m of water for eight months on the standard recurrent cycle, as well as summer floods in both years.

**Table 5:** Comparison of total established sites number in twelve sampling sites of eight woody plant species in both 2009 and 2010.

Species	2009	2010
*Vitex negundo* L.	8	2
*Morus alba* L.	6	1
*Sapium sebiferum* L. Roxb.	6	2
*Glochidion puberum* L. Hutch.	5	1
*Rhus chinensis* Mill.	4	1
*Melia azedarach* L.	3	0
*Pterocarya stenoptera* DC.	3	1
*Trema levigata* Hand.-Mazz.	3	2

### Patterns of life form, species richness, community diversity and above-ground biomass along elevational gradient

**Figure 3:**
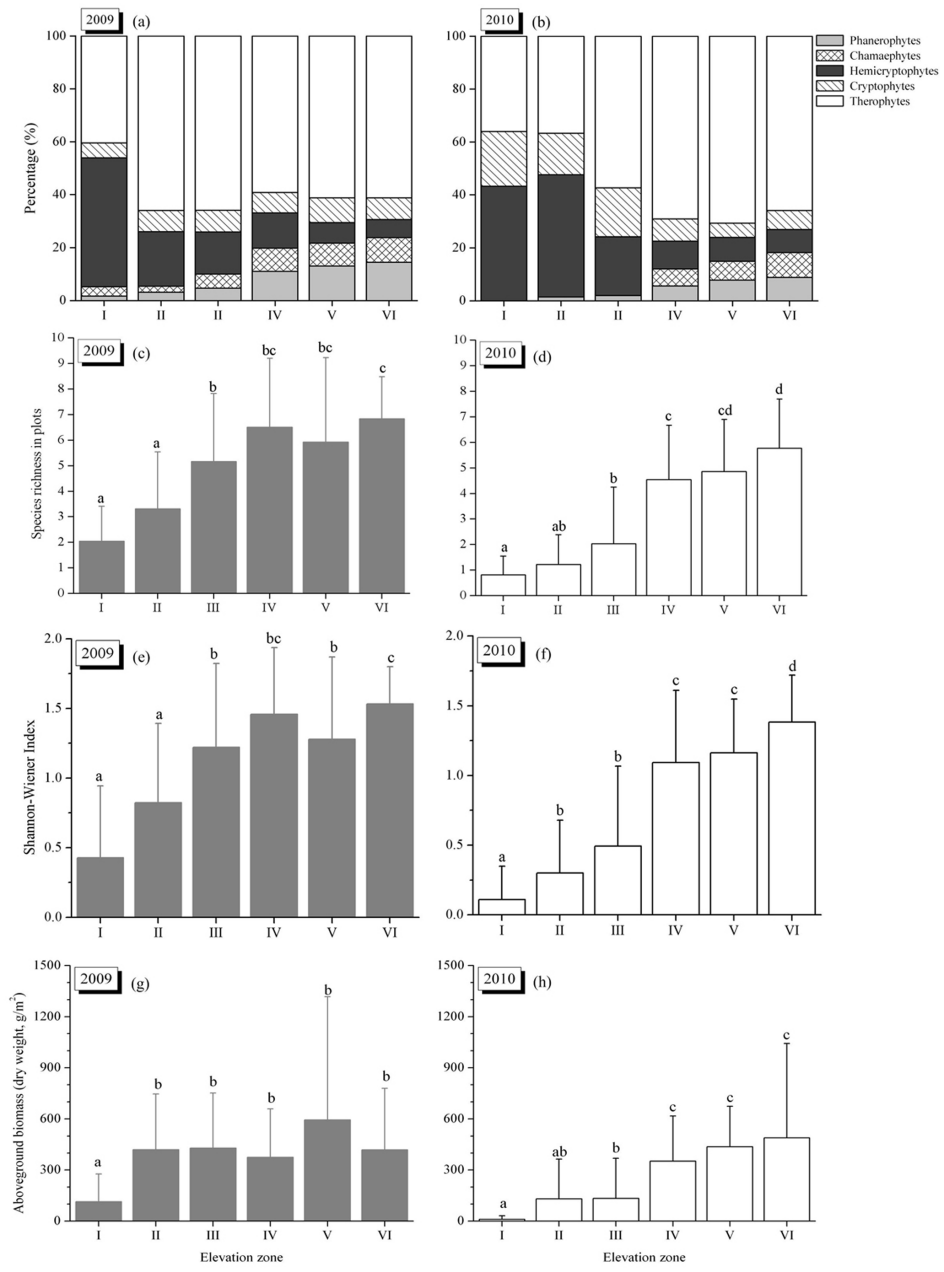
Life form percentage, species richness in plot (mean±SD), Shannon-Wiener Index (mean±SD) and above-ground biomass (mean±SD) of vegetation communities across the elevation zones in 2009 and 2010. Life form percentage was calculated by total recorded plant species date in each elevation zone. Different letters (a, b, c) above the boxes indicate significant differences for *P*<0.05.

Differences of the compositions of plant life-forms across the elevation zones were evident in both years (Fig. 3 a, b). In 2009, therophytes were the dominant life form in all elevation zones except zone I, with proportions ranging from 40.4% in zone I to 66.0% in zone II. Hemicryptophytes dominated zone I at 48.8%. The proportion of phanerophytes increased steadily with elevation from 1.7% in zone I to 14.5% in zone VI. The proportion of cryptophytes was relatively constant across the elevation zones ranging from 5.6% in zone I to 9.3% in zone V. The lowest incidence of chamaephytes was in zone II (2.2%) and the highest in zone VI (9.3%). In 2010, therophytes and hemicryptophytes were still the dominant life forms and their proportions were relatively constant (around 80%) across the elevation zones. Compared with 2009, the proportions of chamaephytes and phanerophytes decreased in all zones and chamaephytes disappeared from the drawdown area at elevations below 160 m.

Analysis of the species richness of the plots in the six elevation zones revealed significant differences in both years (*P*<0.05, Fig. 3 c, d). In 2009, zone I kept a lowest species richness of 2.0±1.4 per plot, while zone IV had the highest (6.8±1.7 species per plot). The species richness of the zones above the elevation of 155 m (zone III to zone VI), which were flooded for the first time in the winter of 2008, was significantly higher (*P*<0.05) than that of zones I and II which had been submerged three times. Mean species richness per plot increased with elevation, except for a slight reduction in zone V running counter to the trend. In 2010, species richness in the plots ranged from 0.8 in zone I to 5.8 in zone VI. The species richness values for all elevation zones were depressed relative to 2009, particularly in the plots from zone II and III with decreases of more than 60%. In 2010, the zones below 160 m were significantly different from those above 160 m (*P*<0.05, Fig. 3 d).

In 2009, Shannon-Wiener index in the six elevation zones lay between 0.4 and 1.5 with an average of 1.1 (Fig. 3 e), while in 2010 it was between 0.1 and 1.3 with an average of 0.8 (Fig. 3 f). The spatial patterns of Shannon-Wiener index in the plots across the six elevation zones, and its annual variation, were generally similar to species richness.

Above-ground biomass patterns across the elevation zones in 2009 (Fig. 3 g) were different from species richness and Shannon-Wiener index. The above-ground biomass in zone I (114.2±162.5 g/m^2^) was significantly different from that of other elevation zones (*P*<0.05), while there were no significant differences among the elevation zones above 150 m (*P*>0.05). In 2010, the biomass in zone I had decreased to 10.2±21.4 g/m^2^ (Fig. 3 h). The biomass of zones II and III that had been affected by summer flooding were reduced to 130.4±234.1 g/m^2^ and 133.0±235.7 g/m^2^ respectively, and were significantly lower than elevation zones above 160 m (*P*<0.05).

### Effect of environmental parameters

Spearman's rank correlation coefficients (*r_s_*) among biodiversity indices, above-ground biomass and environmental factors are presented as a matrix in Table 6. Significant positive correlations were found among in-plot species richness, Shannon-Wiener index, above-ground biomass and elevation in the two years. Both submergence duration in the previous winter and submergence duration in the summer had significant negative correlations with the vegetation community biodiversity index and above-ground biomass. Furthermore, their *r_s_* were higher in 2010 than in 2009. Most of the Spearman rank correlations between the other four environmental factors with either biodiversity index or above-ground biomass were lower indicating their relatively weak influences on the vegetation in the drawdown area (Table 6).

**Table 6:** Spearman rank correlation matrix (*r_s_*) among biodiversity indices, above-ground biomass and environmental factors.

2009	R	H′	Ab	Ele	Sub	Smc	Sl	Sdw	Sds
R	1.00								
H′	0.96**	1.00							
Ab	0.24*	0.31**	1.00						
Ele	0.67**	0.64**	0.32**	1.00					
Sub	0.26*	0.23	−0.17	0.18	1.00				
Smc	−0.12	−0.11	0.41**	−0.23	−0.17	1.00			
Sl	0.22	0.17	−0.06	0.04	0.06	0.22	1.00		
Sdw	−0.67**	−0.64**	−0.32**	−1.00**	−0.18	0.23	−0.04	1.00	
Sds	−0.60**	−0.58**	−0.46**	−0.65**	−0.16	0.07	−0.04	0.65**	1.00

2010	R	H′	Ab	Ele	Sub	Smc	Sl	Sdw	Sds
R	1.00								
H′	0.98**	1.00							
Ab	0.70**	0.71**	1.00						
Ele	0.78**	0.81**	0.67**	1.00					
Sub	0.20	0.19	0.01	0.19	1.00				
Smc	0.06	0.01	0.18	−0.22	−0.15	1.00			
Sl	0.02	0.03	0.03	0.04	0.14	0.16	1.00		
Sdw	−0.78**	−0.81**	−0.67**	−1.00**	−0.19	0.22	−0.04	1.00	
Sds	−0.79**	−0.81**	−0.71**	−0.94**	−0.19	0.16	−0.05	0.94**	1.00

*P<0.05,

**P<0.01.

R: species richness in plots, H: Shannon-Wiener Index, Ab: above-ground biomass, Ele: elevation, Sub: substrates, Smc: soil moisture content, Sl: slope, Sdw: duration of submergence in previous winter, Sds: duration of summer submergence.

**Figure 4:**
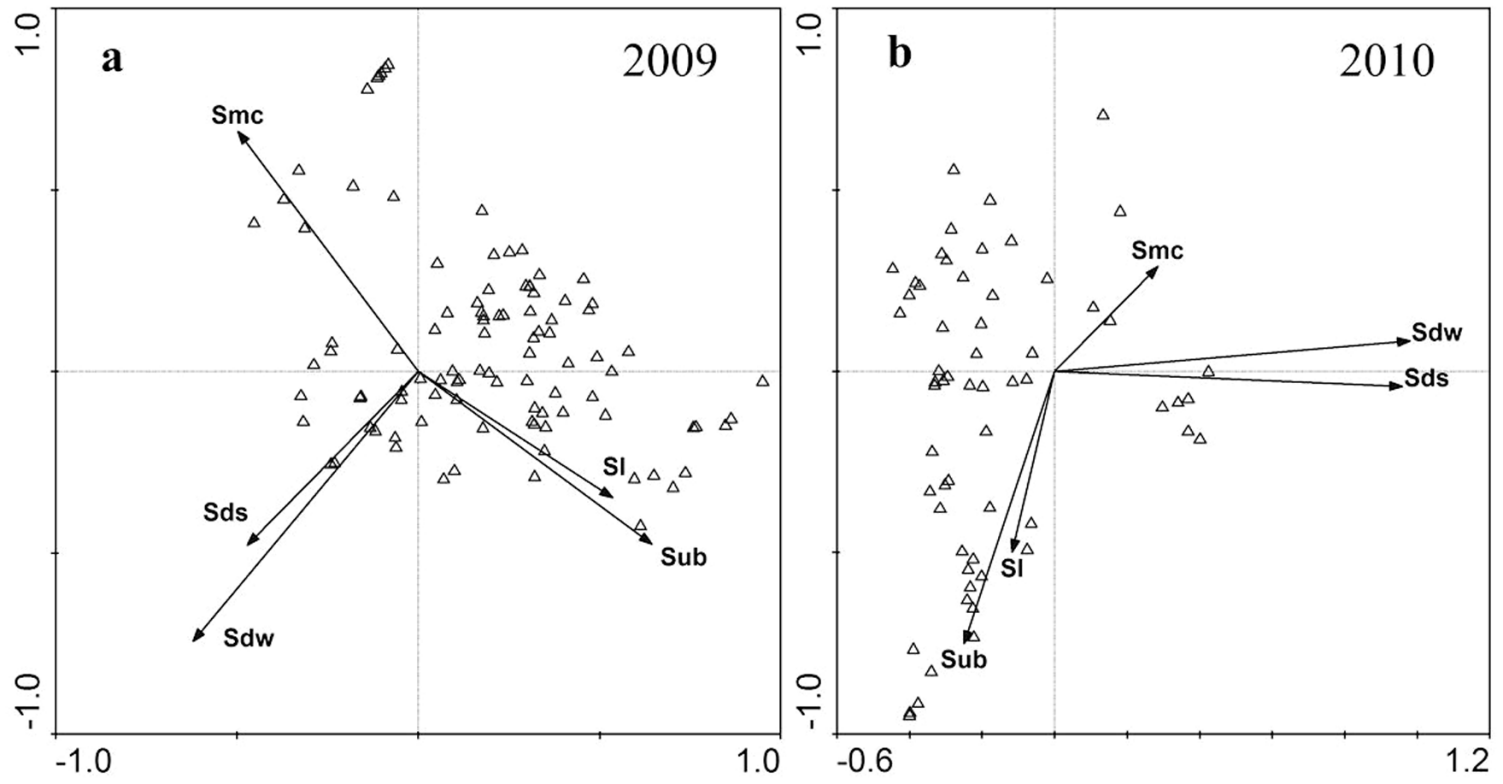
CCA diagram for the relationship between environmental variables and plant species in the DATGR in 2009(a) and 2010 (b). Abbreviations see Table 6.

Relationship between plant species and environmental variables was significant (Table 7). The first and second CCA axis together explained more than 60% variation in vegetation in both years (Table 7). Comparing CCA results of 2009 and 2010, however, a difference between the relationship of vegetation and environmental variables would be found (Table 7, Fig 4). In 2009, the first axis was related to substrate and the second axis indicated a gradient of duration of submergence in previous winter. While, in 2010, the first axis represented a gradient of both duration of submergence in previous winter and duration of summer submergence. The second axis reflected the gradient of substrate.

**Table 7:** Results of CCA for the relationship between environmental variables and vegetation in the DATGR in 2009 and 2010.

	2009		2010	
	Axis1	Axis2	Axis1	Axis2
Eigenvalues	0.566**	0.460**	0.602**	0.275**
Species-environment correlation	0.845	0.810	0.865	0.679
*Cumulative percentage variance*				
of specie date	3.1	5.7	5.1	7.4
of species- environment relation	33.7	61.0	45.9	66.9
*Correction with first two axes*				
Sds	−0.3988	−0.3876	0.8295	−0.0282
Sdw	−0.5250	−0.6025	0.8478	0.0566
Sub	0.5442	−0.3856	−0.2166	−0.5098
Smc	−0.4204	0.5355	0.2467	0.1956
Sl	0.4530	−0.2819	−0.1009	−0.3386

**P<0.01.

Significance of the first two ordination axes was assessed by Monte Carlo permutation testes (9999 permutations). Abbreviations see Table 6.

## Discussion

### Vascular flora biodiversity loss in the drawdown area

The DATGR lies in a region considered to be one of the three richest centers of floral diversity in China [15] and also recognized as one of 25 biodiversity hotspots in the world [16] due to its unique geography and complex topography [7]. Zhong [17] reported about 392 vascular plant species in the pre-dam riparian vegetation in the area, now the DATGR. However, only 231 vascular plant species were found there in 2009 [18] demonstrating that almost 43.0% of the vascular flora had disappeared or become rare in the drawdown area after the first impoundment to 172 m in the winter of 2008. We found 27.4% of species disappeared in the sampling sites in 2010 compared with 2009. This remarkable ongoing decline of floral diversity indicates the effective influence of deep and long duration flooding on eliminating species (Table 6), given that more than 80% of the drawdown area was riparian upland of the Yangtze River and its tributaries [14] and the flora there were seldom if ever inundated by flooding before the construction of the TGD [13]. Our results continue to confirm the former prediction that the new water regime of the TGR would give rise to reduction in quantity and diversity of vegetation [7].

**Figure 5:**
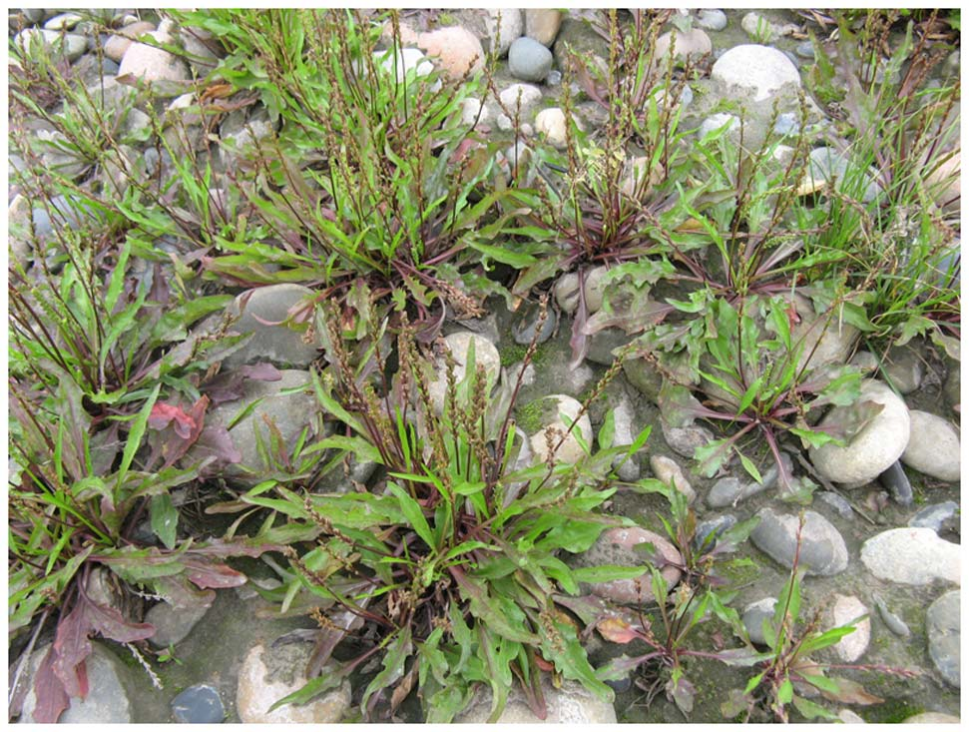
*Plantago fengdouensis* on one side bar at the head of the Three Gorges Reservoir. Photo by Qiang Wang.

Nevertheless we cannot ignore that as a consequence of thousands of years of human activity (including farming, deforestation, urbanization, and so on) the pre-dam vegetation in the DATGR was dominated by herbs and shrubs, and forest was secondary or artificial ([19]–[21]. Almost a third of the pre-dam flora consisted of widely distributed annual species [17]. Pre-dam research found that the habitat of only two endemic plant species (*Myricaria laxiflora* (Franch.) P. Y. Zhang et Y. J. Zhang and *Plantago fengdouensis* (Z.E.Zhao et Y.Wang) Y.Wang et Z.Y.Li) would be completely submerged in the TGR [22], [23]. The former was distributed in the pre-dam riparian zone of the mainstream of the Yangtze River [24], while the latter was located on its three central bars in Chongqing [22, 23). However, field populations of *Myricaria laxiflora* were also found in 2007 on three central bars of the Yangtze River downstream of the TGD [24] and recently a large amount of *Plantago fengdouensis* was found on a side bar at the head of the TGR (Fig. 5). Therefore, in spite of serious floral decline in the drawdown area, there seem to have been no plant extinctions in the TGRA as a direct result of the impoundment of the TGR.

Assessing the life-form spectra of plant communities is a means of classifying vegetation by taking account of the position and degree of protection of the buds from which plant growth is renewed (see for example [25]). We found that plants with highly protected buds (therophytes and cryptophytes) are becoming the only plant life-forms able to persist in the most deeply flooded parts of the drawdown area (zones I and II). By contrast, the full range of plant life-forms are tending to persist at higher elevations (zones IV–VI) where flooding is relatively shallow and short-lived. While therophytes are also abundant in zones IV–VI, plants with relatively exposed buds (phanerophytes and chamaephytes) also persist there. It is generally accepted that species having buds that are more protected are better able to survive harsh conditions during unfavourable seasons, in this case winter flooding. Life-form will therefore be a useful criterion for the purpose of selecting plants suited to the conditions in the various zones of the drawdown area

### Flood-tolerant perennial plants

Tolerance of riparian plants to natural flooding is variable [6], [26]–[30]. In order to find plants for vegetation restoration in the drawdown area, laboratory experiments have been conducted to test the flooding tolerance of local riparian plants by simulating the changing soil water conditions of the TGR. From these experiments some perennial plants were suggested as potential choices, such as *Salix variegate* [31], *Arundinella anomala* Stend. [32], *Pterocarya stenoptera* [33] and *Morus alba* [34]. However, our current investigation found these plants cannot persist well in the hydrological regime characterized by prolonged winter impoundment and short-term summer flooding. We found by 2009 surviving trees and shrubs were distributed only in areas above 160 m. The difference between the field observations and the outcome of laboratory experiments may be explained by the fact that only a single winter of flooding, or summer flooding, were considered in those experiments. It appears that repeated exposure to the environmental stresses in the TGR is more severe than a single exposure.

In this study *Cynodon dactylon* and *Cyperus rotundus* were found to have high tolerance to the new water regime in the TGR. Our field observations confirm earlier laboratory experiments [35], [36]. Together, these two species alone formed a distinctive high density vegetation community in lower elevation regions of the drawdown area. It appears that *C. dactylon* and *C. rotundus* will be valuable for soil conservation in bare soil areas [13].

### Influence of environmental factors

Natural riparian areas commonly display plant zonation that follows an elevation gradient consistent with soil moisture conditions [37]. Our research indicated there were obvious variations in plant biodiversity and above-ground biomass along the elevation gradient of the drawdown area. The effect of soil moisture seems not as important, however, as that in natural riparian zones (Table 6, Table 7). There may be two possible explanations for this observation. First, seasonal flooding of the TGR decreases the capillary porosity and aeration capability of the soil [38]. As a result, the soil in the drawdown area does not hold water and air for plant growth as easily as it did previously. The second reason may be more important. The soil in our sampling sites was generally arid during the whole summer due to the effects of summer drought and high temperatures, except in lower elevation zones where summer flooding saturates the soil for short periods.

**Figure 6:**
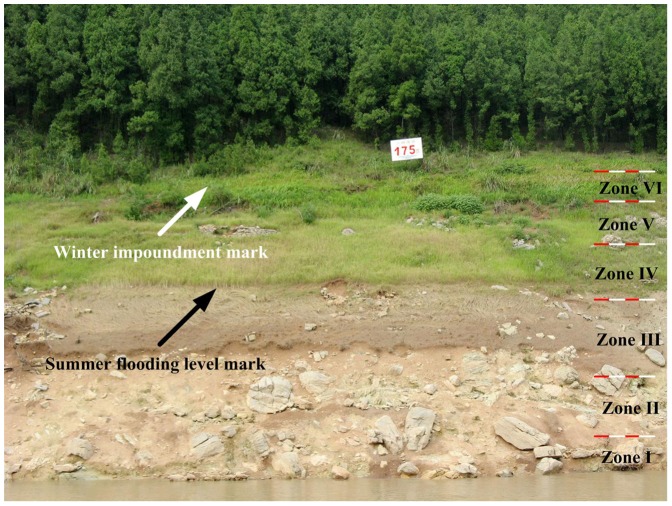
An example of the drawdown area of the Three Gorges Reservoir beside the mainstream of the Yangtze River in Badong County, Hubei Province, on August 2010, showing unaltered vegetation above the 175 m high water mark, summer growth of vegetation between the winter and summer flooding marks, and the bare ground resulting from alternating summer flooding at the lowest level. Approximate superimposed sampling elevation zones were marked to exhibit relative position between them and the flooding of winter and summer. Photo by Qiang Wang.

Little research has been focused on the effects of winter flooding on vegetation in the drawdown area of reservoirs until the impoundment of the TGR [39]–[42]. One reason for this is that only a few very large reservoirs are subjected specifically to winter flooding regimes. In China, the Xiaolangdi Reservoir and the Sanmenxia Reservoir on the Yellow River are the other two very large reservoirs besides the TGR in which the water level is kept high in winter. Knowledge of the effects of winter flooding will not be readily obtained from examining the vegetation in the drawdown area of reservoirs subjected to summer flooding as this affects plants differently. Flooding during the prime growing period reduces light and oxygen availability to plants, limits the growth season, and may prevent annual species from completing their life cycle [7]). The drawdown area of summer-flooded reservoirs is immaturely developed and relatively barren [43]. In our research we have found that an exposure period of four months is sufficient for fast growing plants to complete their life cycles and for plant communities to develop or be maintained. The CCA indicate that most of plant species distributed in the area with less winter submergence duration (Fig. 4) and vegetation cover in higher elevations of the drawdown area is extensive (Fig. 6). The difference between the vegetation in the drawdown area of most summer flooded reservoirs by comparison with the TGR indicates the importance of timing in shaping riparian plant communities.

Flooding tolerance of riparian species can be expected to vary according to the season in which flooding is experienced. Riparian species have been reported to be less tolerant of flooding in summer than in winter, and tolerance to summer flooding has been shown to explain the distribution of plant species along elevation gradients in floodplains [44]. Dramatic loss of plant species in floodplains can be caused by extreme summer flooding and recovery of the vegetation has been reported to take several years [45]. Van Eck et al. [44] therefore suggested that summer flooding had much more impact than winter flooding on species distribution in a floodplain. According to our analysis (Table6, Table 7), the effect of summer flooding of the Yangtze River on vegetation in the drawdown area was not as significant as that of winter impoundment of the TGR in2009. In 2010, however, the effect of summer flooding was quite similar to that of winter impoundment basing on correction with first two ordination axes and Spearman's rank correlation coefficient. The variation may be explained by the difference between magnitude and duration of summer flooding in 2009 and 2010. In the first year, the summer flooding just rose a few meters and kept only thirteen days, while in next summer it rose up to 160 m in elevation and submerged I zone more than one and a half months (Table 3, Fig. 2). In spite of this, in our research it was still difficult to identify which flood period (summer or winter) had more influence on the vegetation. One reason is that duration (half year on average *versus* two months), magnitude (30 m *versus* about 15 m at maximum) and frequency (once per year *versus* several times in summer) were quite different between winter and summer flooding in the TGR. As almost half of the flora in the drawdown area consists of annual species, there are important differences in the status of the species that are influenced by the flooding in winter versus summer. Winter flooding selected for seeds that tolerated long-term winter submersion, while summer flooding selected for plantlets that withstood extreme flooding during their growth season.

### Environmental consequences

The vegetation in the DATGR is a vital part of ensuring the functional life of the dam, connectivity of the surrounding ecosystems, preservation of local biodiversity, health of the watershed [7], decrease of soil erosion and removal of pollutants [13]. However, decomposition of vegetation after winter impoundment can cause environmental problems in the following spring, such as emission of greenhouse gases, decline of water quality, accumulation of methylmercury, eutrophication of the water body, and algal blooms. The average above-ground biomasses of our twelve sampling sites were about 391.7 g/m^2^ and 258.4 g/m^2^ in 2009 and 2010, respectively. Considering the area suggested by Zhang [14], we estimate that as much as several million tonnes of vegetation biomass would be accumulated in the DATGR every year. The vegetation is submerged every winter and its total amount must be much more than that of the former riparian fringe beside the Yangtze River and its tributaries. In the TGRA, as the former riparian zone was mostly narrow and steep with little vegetation cover [46]. Currently we know little about the underwater decomposition of the vegetation following winter impoundment, and how this will influence the environment of the TGR, as no research on this has been reported yet. It can be speculated that the problem introduced by rotting of vegetation may be not be as serious as that of tropical reservoirs (e.g. Tucuruí Reservoir in Brazil [47] because woody vegetation was cleared before construction of the TGD and the colder water of the TGR during winter lowers the decomposition rate. There is still reason for concern however, as year-upon-year accumulation of dead vegetation in the TGR may change the situation. Therefore, it will be important to study the decomposition of winter-submerged vegetation and the associated environmental consequences.
