# Correction: Diversity and Above-Ground Biomass Patterns of Vascular Flora Induced by Flooding in the Drawdown Area of China's Three Gorges Reservoir

**DOI:** 10.1371/journal.pone.0147452

**Published:** 2016-01-19

**Authors:** Qiang Wang, Xingzhong Yuan, J. H. Martin Willison, Yuewei Zhang, Hong Liu

This study is a follow up to an earlier study reporting the results of a survey of vegetation carried out in 2009 which was published in the *Polish Journal of Ecology* (Reference 13 in the published article):

Wang Q, Yuan XZ, Liu H, Zhang YW, Cheng ZL, et al. (2012) Effect of long-term winter flooding on the vascular flora in the drawdown area of the Three Georges Reservoir, China. Polish Journal of Ecology 60: 95–106.

The current article reported a comparison of two consecutive years of data (2009 and 2010) using data from 12 of the 14 sites included in the original study; thus, some of the data generated by the 2009 survey and used in this analysis has been previously published. The relationship of the current study to the previous work was acknowledged in the paper; however, the authors would like to provide clarification of a number of instances in which previously published information/data were used in the article without an accompanying clear citation:

- Information on sampling methods and the hydrological regime was similar to that reported in the *Polish Journal of Ecology* article.
- The 2009 data on life forms (shown in Table 4) for 12 of the 14 original sampling sites were re-used from the previous study.
- The 2009 data on patterns of life form and species richness along elevational gradient for 12 of the 14 original sampling sites were re-used from the previous study.

In addition, the authors wish to clarify that Figs 1 and 3A were modified from the above article with permission from copyright holder *Polish Journal of Ecology*. The captions for Figs 1–3 and Table 2, Table 4 and Table 5 should be revised to read as follows:

**Fig 1:**
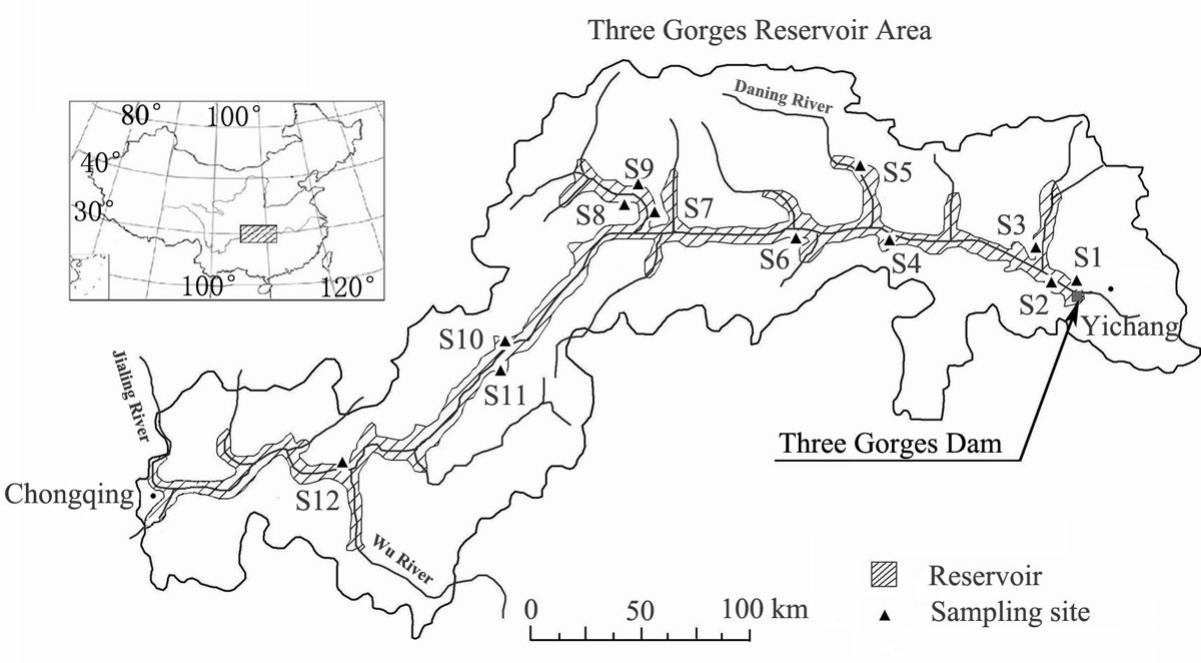
Spatial distribution of the twelve sites sampled in 2010. The sites sampled in 2009 were mostly the same and were mapped previously [13]. The Three Gorges Reservoir Area is a combination of all the counties around the reservoir. Modified from [13] with permission from *Polish Journal of Ecology*.

**Fig 2:**
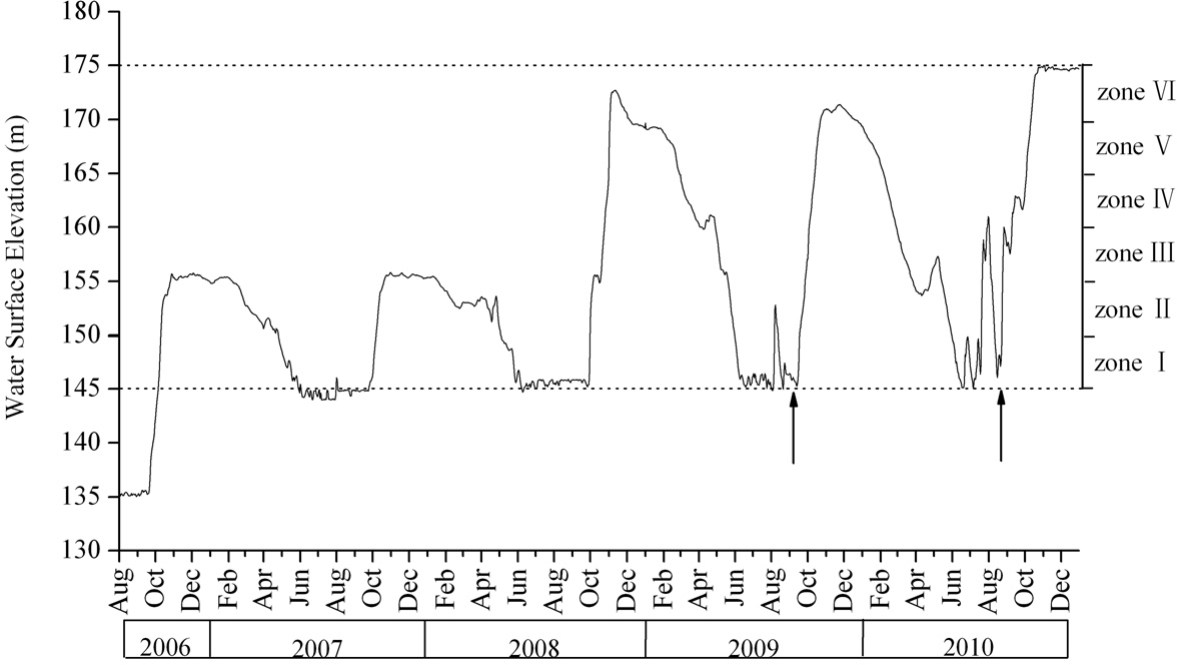
Water level of the Three Gorges Reservoir between August 2006 and December 2010. Data updated from [13] to include July 2009 –Dec 2010. Data originate from the daily records of the Three Gorges Dam hydrology station (http://www.ctgpc.com.cn/index.php). The vertical arrows indicated the period for field sampling.

**Fig 3:**
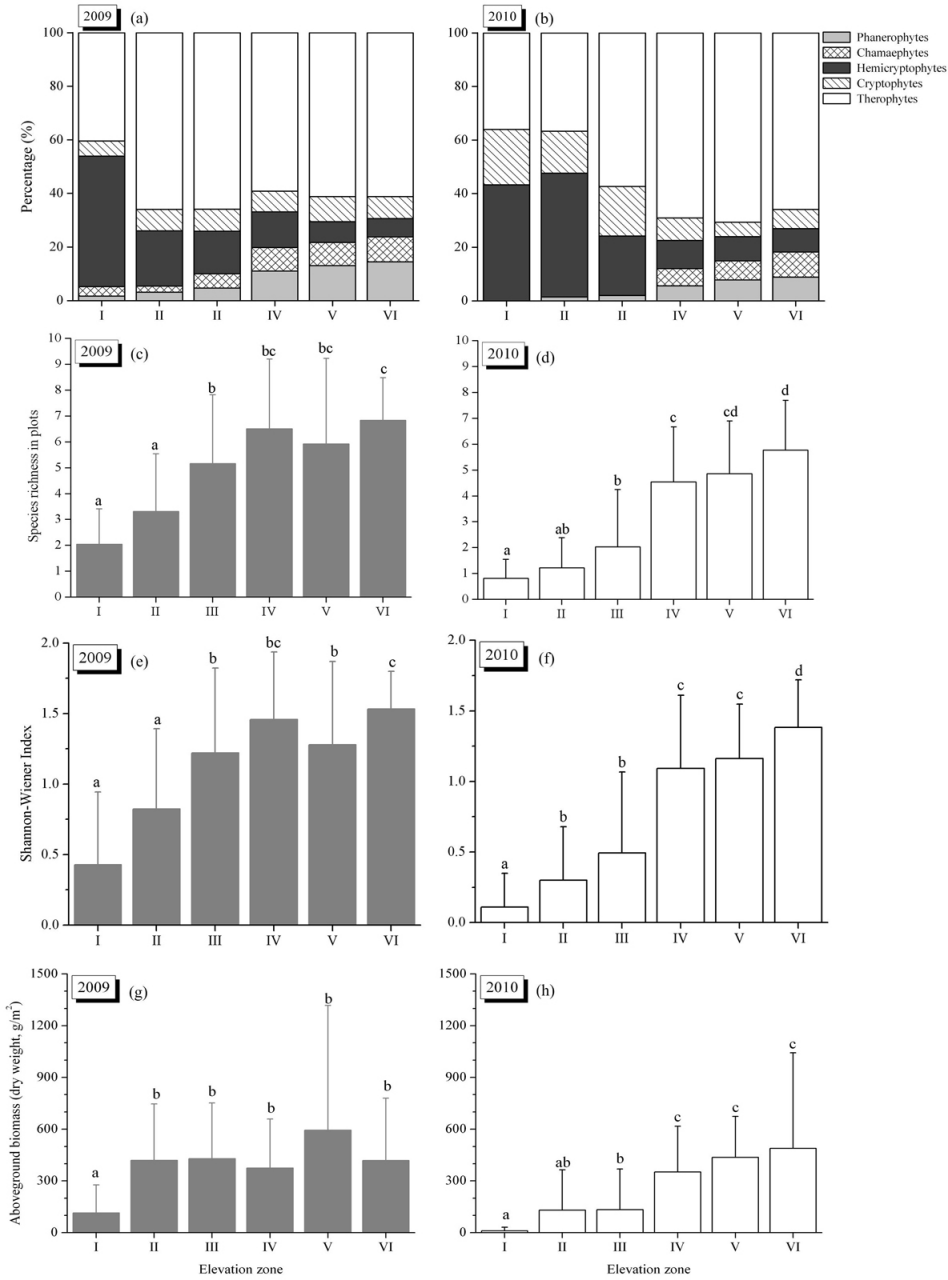
Life form percentage, species richness in plot (mean±SD), Shannon-Wiener Index (mean±SD) and above-ground biomass (mean±SD) of vegetation communities across the elevation zones in 2009 and 2010. Life form percentage was calculated by total recorded plant species date in each elevation zone. Different letters (a, b, c) above the boxes indicate significant differences for *P*<0.05. 3(a) is modified from [13] with permission from *Polish Journal of Ecology*. Life form percentage and species richness data for 2009 are taken from 12 of the 14 original sampling sites as previously reported in [13].

**Table 2:** General environmental characteristics of the twelve sampling sites in the drawdown area of the Three Gorges Reservoir, as previously reported in [13].

Sampling site	Location	Longitude/latitude	Slope	Pre-dam land use
S1	Taipingxi in Zigui county	30°51′N, 110°59′E	25°	Dry land with a few woods
S2	Xiaoxingtan in Xiling Gorge	30°56′N, 110°46′E	34°	Shrubs and dry land
S3	Xiangxi River Estuary	30°58′N, 110°45′E	15°	Dry land
S4	Luyou Hole in Wu Gorge	31°03′N, 109°54′E	48°	Shrubs
S5	Dachang in Wushan County	31°15′N, 109°49′E	20°	Dry land
S6	Baidicheng in Qutang Gorge	31°02′N, 109°34′E	25°	Shrubs
S7	Shuangjiang in Yunyang County	30°56′N, 108°41′E	30°	paddy field
S8	Baijia Stream in Kaixian County	31°08′N, 108°33′E	15°	Rice paddy and dry land
S9	Laotudi in Kaixian County	31°09′N, 108°34′E	10°	Paddy field
S10	Huanghua Island in Zhongxian County	30°19′N, 108°05′E	20°	Paddy field
S11	Dongxi Stream in Zhongxian County	30°16′N, 108°04′E	18°	Paddy field
S12	Baiyansi in Fuling County	29°43′N, 107°23′E	25°	Dry land and village land

**Table 4:** Comparison of the absolute number and percentage of each life form category in the drawdown area before impoundment by the Three Gorges Dam and after impoundment in both 2009 and 2010. Data for 2009 are taken from 12 of the 14 sites as previously reported in [13].

Life form	No. of sp.		Percentage (%)	
	2009	2010	2009	2010
Phanerophytes	30	16	17.1	12.6
Chamaephytes	14	11	8.0	8.7
Hemicryptophytes	16	14	9.1	11.0
Cryptophytes	28	23	16.1	18.1
Therophytes	87	63	49.7	49.6
Total	175	127	100	100

**Table 5:** Comparison of total established sites number in twelve sampling sites of eight woody plant species in both 2009 and 2010. Data from 2009 survey for *Vitex negundo* L., *Morus alba* L., and *Sapium sebiferum*L. Roxb. as previously reported in [13].

Species	2009	2010
*Vitex negundo* L.	8	2
*Morus alba* L.	6	1
*Sapium sebiferum* L. Roxb.	6	2
*Glochidion puberum* L. Hutch.	5	1
*Rhus chinensis* Mill.	4	1
*Melia azedarach* L.	3	0
*Pterocarya stenoptera* DC.	3	1
*Trema levigata* Hand.-Mazz.	3	2
